# Impact of Equal Channel Angular Pressing on Mechanical Behavior and Corrosion Resistance of Hot-Rolled Ti-2Fe-0.1B Alloy

**DOI:** 10.3390/ma13225117

**Published:** 2020-11-13

**Authors:** Yanhuai Wang, Xin Li, I. V. Alexandrov, Li Ma, Yuecheng Dong, R. Z. Valiev, Hui Chang, Biao Zhang, Yuyang Wang, Lian Zhou, Zhiwei Hu

**Affiliations:** 1 College of Materials Science and Engineering/Tech Institute for Advanced Materials, Nanjing Tech University, Nanjing 211816, China; 201861203145@njtech.edu.cn (Y.W.); 201961203183@njtech.edu.cn (B.Z.); 201961103041@njtech.edu.cn (Y.W.); zhoul@c-nin.com (L.Z.); 2 Jiangsu Collaborative Center for Advanced Inorganic Function Composites, Nanjing Tech University, Nanjing 211816, China; 3 State Key Laboratory for Marine Corrosion and Protection, Luoyang Ship Material Research Institute (LSMRI), Qingdao 266237, China; mal@sunrui.net; 4 Huaneng Fuzhou Power Plant, Fuzhou 350200, China; xinge@njtech.edu.cn; 5 Department of Physics, Ufa State Aviation Technical University, 450008 Ufa, Russia; igorvalexandrov@yandex.ru; 6 Institute of Physics and Advanced Materials, Ufa State Aviation Technical University, 450008 Ufa, Russia; ruslan.valiev@ugatu.su; 7 College of 2011, Nanjing Tech University, Nanjing 211816, China; 4201170215@njtech.edu.cn

**Keywords:** equal channel angular pressing, ultrafine-grained, Ti-2Fe-0.1B alloy, mechanical behavior, corrosion resistance

## Abstract

In the present study, the unique bimodal grain size distribution microstructure with the ultrafine substrate and embedded macro grains was fabricated by a traditional hot-rolling process in a novel low-cost Ti-2Fe-0.1B titanium alloy, which possesses a good combination of strength (around 663 MPa) and ductility (around 30%) without any post heat treatment. Meanwhile, the mechanical behavior and corrosion resistance of hot-rolled Ti-2Fe-0.1B alloy after equal channel angular pressing (ECAP) deformation were studied. Results indicated that the average grain size decreased to 0.24 μm after 4 passes ECAP deformation, which led to the enhancement of tensile strength to around 854 MPa and good ductility to around 15%. In addition, corrosion resistance was also improved after ECAP due to the rapid self-repairing and thicker passivation film. Our study revealed that the novel low-cost titanium alloy after hot-rolling and ECAP could be used instead of Ti-6Al-4V in some industrial applications due to similar mechanical behavior and better corrosion resistance.

## 1. Introduction

Titanium and its alloys are widely used in aerospace, marine industry and biomedical fields due to their high specific strength, excellent fatigue strength and good compatibility [1,2,3]. However, the high cost of titanium alloy is the fatal drawback that seriously restricts its wider application. As the strongest eutectoid β stabilizing element, Fe is constantly used to design low-cost titanium alloys due to its cheap price, which could also improve mechanical properties simultaneously through the solid solution strengthening effect [4,5,6]. For instance, Louzguine et al. [5] investigated the mechanical behavior of Ti-1Fe (wt.%) and Ti-3Fe (wt.%) alloys, a wide range of mechanical properties from high strength (1.3 GPa) and low ductility (2%) to intermediate strength (700 MPa) and high ductility (30%) was achieved by proper heat treatment. Meanwhile, our previous work also studied the effect of Fe content on microstructure evolution and mechanical properties of as-cast Ti-xFe-0.1B (x = 1~5, wt.%) alloy [6], we found that the alloy can achieve the best match between strength and plasticity with greater potential for development when the Fe content is in the range of 3 wt.%~4 wt.%. However, more element Fe addition can lead to segregation and decline of the properties of titanium alloy, especially for large ingot production at the industrial level [7]. Therefore, the content of Fe is always controlled less than 3% for most Titanium alloys [8,9].

In recent decades, severe plastic deformation (SPD) has been used to produce ultrafine-grained (UFG)/nanostructure (NS) materials and improve their physical and chemical properties [10,11,12]. Among them, equal channel angular pressing (ECAP) is one of the most effective methods which can be used to fabricated bulk UFG materials and has great industrial application potential [13,14]. Many UFG metals including Titanium fabricated by ECAP exhibit improved mechanical properties, for example, the high strength of 627 MPa and 1435 MPa could be achieved after ECAP for Grade 2 [15] and Grade 5 [16] Titanium alloys respectively. At the same time, considering a wide application in the field of chemical, biomedical and marine engineering, the study of corrosion behavior is very essential for titanium alloys. More and more reports have revealed that UFG alloys can achieve excellent corrosion resistance although they contain an increased volume of grain boundaries and dislocations [17,18,19].

Presented in this paper are enhanced mechanical properties and corrosion resistance of a novel low-cost Ti-2Fe-0.1B Titanium alloy processed by hot-rolling, and consequent ECAP supports the point of view that the mentioned Ti-6Al-4V behavior-like titanium alloy could be attractive for some industrial applications.

## 2. Materials and Experimental Procedures

### 2.1. Materials

**Figure 1:**
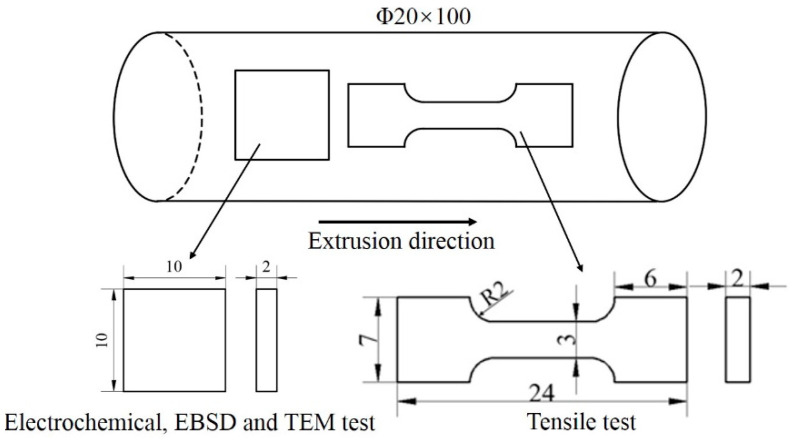
Schematic diagram of sample selected plane for experiments (mm).

Based on the early studies of the research group, a novel low-cost Titanium alloy Ti-2Fe-0.1B, the chemical composition is 1.89% Fe, 0.08% B, 0.014% C, 0.0012% H, 0.062% O, 0.004% N and balance of Ti, was selected to be investigated in present work. The initial materials were mixed and then melted with a vacuum arc remelting furnace (LGX11A-3, Shenzhen, China) by two times, the diameter of ingot casting is 420 mm. The first forging process (cogging, upsetting and drawing-out) made the diameter decrease to ~300 mm at the temperature range of 1020~1050 °C. Then, the precision forging was performed at 900~950 °C to make the diameter of the sample decrease to 125 mm. The sample was finally machined to a diameter 120 mm as a result of peeling. The subsequently rolling process was performed at 836 °C (initial temperature) adopted a 20-stand continuous rolling process. The rolling deformation goes through the alternating multi-pass rolling of horizontal and vertical mills. The sample was rolled into a bar with the diameter from 120 mm to 20 mm gradually (hereafter named “hot-rolled”). Before ECAP, heat treatment was carried out at 780 °C for 1h in a furnace then air cooling (hereafter named “annealed”) was completed. Then, the four ECAP passes were carried out by route Bc at 400 °C using a die with an intersecting channel angle of 90° (hereafter named “ECAPed”). Figure 1 shows the schematic diagram of sample for experiments.

### 2.2. Microstructure Characterization

The microstructure of Ti-2Fe-0.1B alloy was characterized by electron backscatter diffraction (EBSD, Aztec 3.2, Oxford Instruments, Abingdon, UK) and transmission electron microscopy (TEM, FEI G2 60-300, Washington, WA, USA). The samples used to EBSD were prepared by electro-polishing in a solution of 5% perchloric acid (Shanghai Lingfeng Chemical Reagent CO., LTD, Shanghai, China) and 95% ethanol (Sinopharm Chemical Reagent Co., Ltd, Shanghai, China) at room temperature for 120 s with a working voltage of 40 V. The EBSD was carried out in the Field Emission Scanning Electron Microscope (SEM, JSM-6700F, Jeol, Tokyo, Japan) equipped with an Oxford Instruments EBSD detector and worked at an accelerating voltage of 20 KV. The step size of the samples was 0.05 μm (ECAPed sample) and 0.2 μm (hot-rolled and annealed samples) with a scanning area of 1600 μm^2^ separately. The HKL Technology Channel 5 system (Oxford Instruments, Abingdon, UK) was used to analyze the EBSD test data. For the TEM investigation, the samples were mechanical grinding to a thickness of ~60 μm and then jet-polishing with an RL-I electrolytic twin-jet in a solution containing 6% perchloric acid (Shanghai Lingfeng Chemical Reagent CO., LTD, Shanghai, China), 34% butanol (Shanghai Lingfeng Chemical Reagent CO., LTD, Shanghai, China) and 60% methanol (Shanghai Lingfeng Chemical Reagent CO., LTD, Shanghai, China) with an accelerating voltage of 20 KV at the temperature range from −15 °C to 0 °C. TEM was performed using a Tian G2 60–300 TEM instrument (FEI G2 60-300, Washington, WA, USA) worked at an accelerating voltage of 300 KV. Surface morphologies of the samples after electrochemical tests were observed by JSM-6510 scanning electron microscope (SEM, JSM-6700F, Jeol, Tokyo, Japan) with an accelerating voltage of 15 kV.

### 2.3. Mechanical Properties

Tensile tests were carried out on the CSS-44100 electronic universal testing machine (CSS-44100, Jinan, China) with a strain rate of 1 × 10^−3^ s^−1^. The samples with a gauge length 10 mm, a width 3 mm (gauge section) and a thickness 2 mm were used. Three samples were tested for each state and the average values of the ultimate tensile strength (σ_s_), yield strength (σ_0.2_) and elongation to failure (A) were statistically calculated.

### 2.4. Electrochemical Measurements

The samples were subjected to potentiodynamic polarization and electrochemical impedance spectroscopy (EIS, CHI660E, CH Instruments, Austin, TX, USA) with a size of 10 mm × 10 mm × 2 mm. The electrochemical performance of the samples was tested with a CHI660E electrochemical workstation (CHI660E, CH Instruments, Austin, TX, USA) in 3.5 wt.% NaCl solution (Shanghai Lingfeng Chemical Reagent CO., LTD, Shanghai, China). Before the test, the surface of each sample was ground with silicon carbide sandpaper and mechanically polished, then ultrasonically cleaned with alcohol. The nickel wire was connected to the electrochemical samples using an electric welder (PM103-3, Zhenjiang, China). Finally, the samples were encapsulated with epoxy resin. Three-electrode systems were used in these electrochemical measurements, i.e., the working electrode (the hot-rolled and ECAPed samples), the reference electrode (saturated Ag/AgCl) and the counter electrode (a piece of platinum). At first, a stable open circuit potential (OCP) for 10 min was been measured and analyzed. Then, EIS was performed at a frequency from 10^5^ Hz to10^−2^ Hz using an AC voltage amplitude of ±10 mV. Finally, potentiodynamic polarization studies were performed using a voltage from −650 mV to 800 mV with a scanning frequency of 2 mV/s.

## 3. **Results and Discussion**

### 3.1. Microstructure Evolution

**Figure 2:**
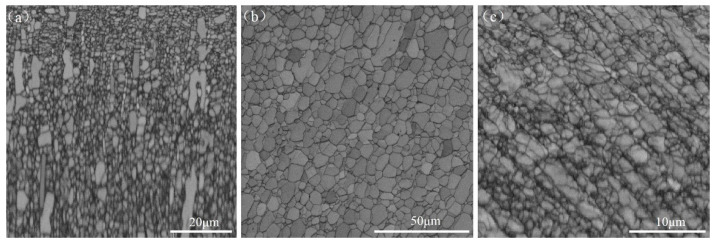
Microstructure of (**a**) hot-rolled, (**b**) annealed and (**c**) equal channel angular pressing (ECAP)ed Ti-2Fe-0.1B alloy as a result of electron backscatter diffraction (EBSD) (note the change of the scale bars).

Figure 2 displays EBSD microstructures of Ti-2Fe-0.1B alloy processed by hot-rolling, annealing and ECAP, respectively. As one can see, the hot-rolled sample is characterized by heterogeneous microstructure, which includes an abundance of ultrafine grains with a grain size less than 1 μm, and micro-grains with grain size approached to 10 μm, as indicated by Figure 2a. It seems like a typical unique bimodal grain size distribution microstructure can be formed directly during the hot-rolling process. A similar phenomenon was also reported by Min Zha et al. [20] in their early research. The average grain size of the hot-rolled sample was calculated as 1.72 μm (Table 1). Figure 2b shows the microstructure of annealed Ti-2Fe-0.1B alloy which is more homogeneous and characterized by the equiaxed grains with the average grain size increased to 3.5 μm. However, the grain size of the sample remarkably decreased to 0.24 μm (Figure 2c) after the consequent ECAP processing. In addition, the grain boundaries tortured severely due to the large accumulated strain in the process of ECAP, the number of subgrains formed with a high density of dislocations led to the grain boundaries interaction and they dim finally. The parameters of high angle grain boundaries (HAGB, θ > 15°), low angle grain boundaries (LAGB, 2° < θ < 15°) and some other relevant parameters of samples at different states obtained through EBSD data statistics are shown in Table 1. 

**Figure 3:**
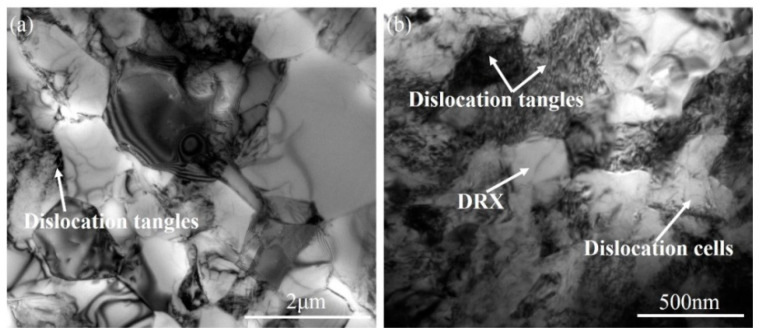
Transmission electron microscopy (TEM) micrographs of (**a**) hot-rolled and (**b**) ECAPed Ti-2Fe-0.1B alloy.

To pursue more detailed information of the microstructure, TEM micrographs of hot-rolled and ECAPed Ti-2Fe-0.1B alloy are presented in Figure 3. The microstructure of the hot-rolled sample mainly consists of nearly equiaxed grains with fewer dislocations and sharp boundaries, although few dislocation pile-ups also can be observed, as shown in Figure 3a. This might be attributed to high initial temperature and heat generation during the high-speed hot-rolling process, which leads to the occurrence of recovery/recrystallization during cooling in air. However, as a result of ECAP, a large volume of dislocations was formed and appeared in not only the grain boundaries but also in the grain interior. The dislocation tangle zones (DTZ) and dislocation cells (DC) can be clearly seen (Figure 3b). However, several dislocation-free grains were also founded in the microstructure, which indicated that continuous dynamic recrystallization started to happen during ECAP with four passes. 

### 3.2. Mechanical Properties

**Figure 4:**
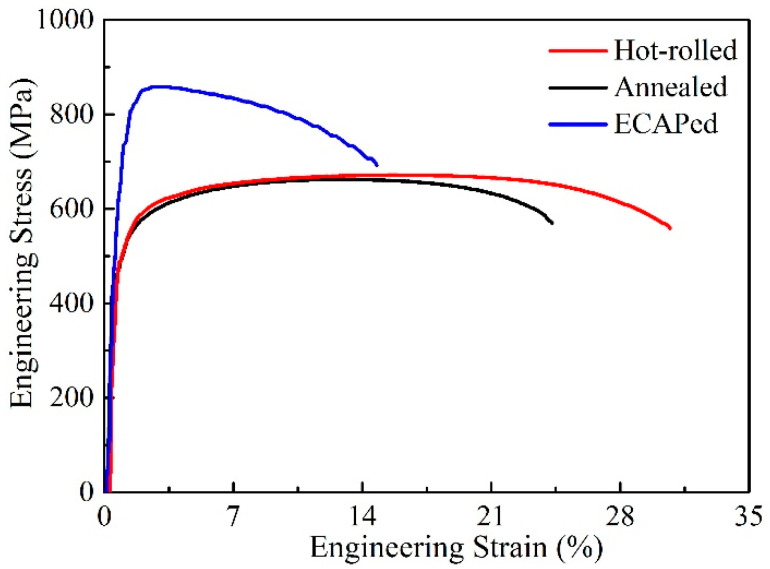
Engineering stress–strain curves of Ti-2Fe-0.1B alloy.

The engineering stress–strain curves of hot-rolled, annealed and ECAPed Ti-2Fe-0.1B alloy are displayed in Figure 4. The yield stress (σ_0.2_) and tensile stress (σ_s_) of hot-rolled sample reach around 457 MPa and 663 MPa, respectively. Furthermore, the elongation to failure of hot-rolled sample can reach around 30%, which indicated a good combination of strength and ductility. On the other hand, after annealing at 780 °C/1 h, the strength of the sample changed slightly, but the ductility decreased to around 24%. The yield stress and tensile stress of the sample increased to around 637 MPa and 854 MPa respectively after the subsequent ECAP process, furthermore, 15% elongation to fracture left. 

It is interesting that the bimodal grain size distribution microstructure can be obtained through the continuous hot-rolling process of the Ti-2Fe-0.1B alloy. It is believed that both the addition of Fe [8] and B [21] elements could seriously refine the microstructure of titanium alloys and induce the formation of ultrafine grain size combined with the effect of large strain during subsequent forging and rolling. Meanwhile, high initial temperature and additional heat generation during the hot-rolling process provided momentary annealing of the Titanium alloy, then led to the unique microstructure distribution with bimodal grain size and possessed a good combination of strength and ductility. However, it is observed that the mechanical behavior of the sample worsened after consequent quasi-static annealing at 780 °C/1 h, especially for the ductility, and the phenomenon was confirmed by plenty of annealing treatments at different temperatures between 600 and 800 °C. Generally speaking, if no harmful phase or particles formed, ductility always improved for the deformed metals subjected to the annealing process due to the increase in grain size or/and decrease in dislocation density. It seems like impurity segregation in grain boundaries [22,23] or/and the formation of ω phase [24] during the annealing process might be responsible for the loss of ductility, and make the strength change slightly. The tensile strength of Ti-2Fe-0.1B alloy increased to around 854 MPa after ECAP, which is already similar to the Ti-6Al-4V. Considering that the average grain size of the sample significantly decreased to ~240 nm after ECAP, based on the Hall–Petch relationship [15,23], it is believed that the fine-grain strengthening effect plays an important role in the increase in the strength in the present work. Furthermore, the increase in dislocation density is also supposed to contribute to the strength of the ECAPed sample. Considering the dynamic recovery and recrystallization during the ECAP process at 400 °C may weaken the effect of distortion strengthening. The strengthening mechanism in the present work is mainly fine grain strengthening and dislocation (grain boundary) strengthening. It is worth noting that the fraction of recrystallized grains is only 2.7% (Table 1) after four passes of ECAP, which is too weak to dominate the mechanical properties of the Ti-2Fe-0.1B alloy after ECAP, and it implies that the mechanical behavior of Ti-2Fe-0.1B alloy could be further improved by improving the microstructure by ECAP.

### 3.3. Corrosion Behavior

**Figure 5:**
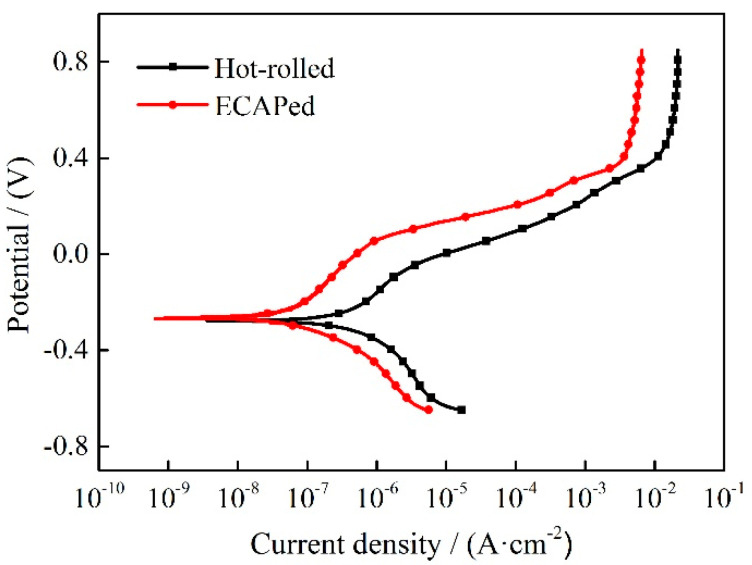
Potentiodynamic polarization curves of hot-rolled and ECAPed Ti-2Fe-0.1B alloy.

The potentiodynamic polarization curves of hot-rolled and ECAPed Ti-2Fe-0.1B alloy in 3.5 wt.% NaCl solution are shown in Figure 5. Results indicate that the two-state samples have the same polarization behavior, and the pitting corrosion occurred in both samples. The phenomenon of passivation was observed before the pitting corrosion occurs. The passivation range of the hot-rolled sample is from −0.286 V to −0.052 V with a passivation region of 0.234 V, while the passivation range of the ECAPed sample is from −0.216 V to 0.065 V with a passivation region of 0.326 V, which is much higher than the hot-rolled state. Meanwhile, the passivation current density (*i*_pass_) of the ECAPed sample is reduced to 0.11 μA·cm^−2^ from 0.82 μA·cm^−2^ in the hot-rolled state. It can be seen that the ECAPed sample is more susceptible to passivation, and the area where passivation occurs is much wider. For the same material, the corrosion rate (*R*) is proportional to the *i*_corr_ and can be computed by Equation (1) [25]:

(1) $$R=3.27\times 10^{-3}\frac{A\times i_{\mathrm{corr}}}{n\rho }$$

where *R* is the corrosion rate (mm/a), *A* is the atomic weight, *i*_corr_ is the corrosion current density (μA/cm^2^), *n* is the chemical valence and *ρ* is the density (g/cm^2^). 

Corrosion behavior of materials can be described by the corrosion potential (*E*_corr_), corrosion current density (*i*_corr_) and other relevant parameters, which can be obtained from the results of the potentiodynamic polarization test. The corresponding parameters of the investigated Ti-2Fe-0.1B alloy are listed in Table 2, which was acquired by Tafel fitting of potentiodynamic polarization curves and calculated by Equation (1). Results indicated that the ECAPed sample has higher *E*_corr_ and pitting potential (*E*_pit_), as well as lower R, *i*_corr_ and *i*_pass_, which means the ECAPed sample is more difficult to corrode and has an excellent corrosion resistance compared with the hot-rolled state. 

**Figure 6:**
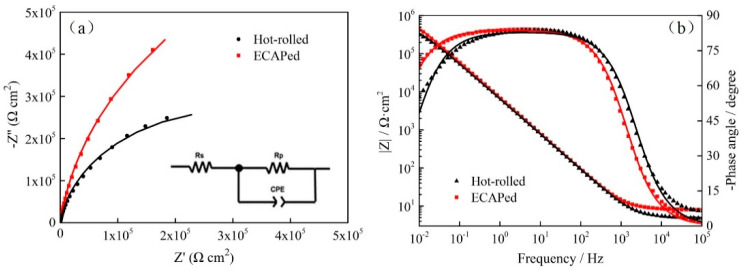
(**a**) Nyquist plots and (**b**) Bode plots for hot-rolled and ECAPed Ti-2Fe-0.1B alloy.

The EIS results of hot-rolled and ECAPed Ti-2Fe-0.1B alloy by the method of Nyquist plots and Bode plots in 3.5 wt.% NaCl solution are shown in Figure 6. The points in the figure represent experimental data, and the solid lines represent electrical equivalent circuit (EEC) fitting data. It can be seen that Nyquist plots are composed of semi-circular arcs for both of the two state samples (Figure 6a). The arc radius of the hot-rolled sample is much smaller than the ECAPed state, which implies that the ECAPed sample has a stronger inhibitory effect on electron transfer and also has a better capacitance characteristic [26,27]. The EEC model used in the present work is inserted into Figure 6a, where the *R*_s_ is solution resistance, *R*_p_ is polarization resistance and CPE is a constant phase element. The CPE reflects the capacitance of the passivation film, which can be calculated using Equation (2).

(2) $$Z_{CPE}={\left[Q{\left(jw\right)}^{n}\right]}^{-1}$$

where *w* represents the angular frequency (rad s^−1^), *Q* is CPE parameter, *j* is the imaginary unit and the factor *n* is defined as the CPE power factor. Additionally, the capacitance of the constant phase element can be measured from Equation (3) [28,29].

(3) $$C=Y_{0}{\left(w_{\max}\right)}^{n-1}$$

where *Y*_0_ is admittance, *w*_max_ represents the angular frequency at which the maximum occurs in the imaginary part of the impedance. In the meantime, the steady-state passivation film thickness (*d*) can be calculated according to Equation (4) [30].

(4) $$d=\frac{\varepsilon \varepsilon_{0}A}{∁}$$

where ε represents the dielectric constant of the passivation film, *ε*_0_ represents the vacuum dielectric constant (8.854 × 10^−14^ F/cm), and A is the effective area.

The electrochemical parameters fitted by EEC are shown in Table 3. The fitting parameter *n* is the CPE index associated with surface roughness and defects, and the value of *n* is usually used to reflect the degree of uniformity of the passivation film [31]. It can be found that the uniformity of the passivation film on the surface of the sample is slightly increased after ECAP processing. The *R*_p_ value is the most accurate response to the corrosion resistance of materials. Generally speaking, the larger value of *R*_p_, the better corrosion resistance of the samples. As can be seen in Table 3, the ECAPed sample has a higher value of *R*_p_ than the hot-rolled sample obviously. Simultaneously, the thickness of the passivation film increased from 1.97 nm in the hot-rolled state to 2.23 nm after the ECAP deformation. The results also indicated that the ECAPed sample possessed better corrosion resistance, which is in accordance with the results obtained from potentiodynamic polarization data in Figure 5 and Table 2.

Regarding the Bode plots, the larger values of |Z| in the low-frequency region indicate better corrosion in samples [32]. As shown in Figure 6b, the low-frequency impedance value (|Z|_0.01Hz_) of the ECAPed sample was higher than that of the hot-rolled sample. On the other hand, the corresponding higher phase angle in the ECAPed sample indicated that the passivation film is more stable and compact on its surface. 

**Figure 7:**
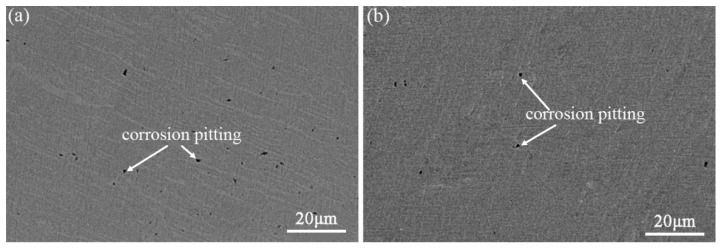
Corrosion morphology of (**a**) hot-rolled and (**b**) ECAPed Ti-2Fe-0.1B alloy after the electrochemical test.

Corrosion morphologies of the hot-rolled and ECAPed Ti-2Fe-0.1B alloy after the electrochemical test are illustrated in Figure 7. It can be found that corrosion pitting happened on the surface both in the hot-rolled and the ECAPed samples. However, the volume of the corrosion pitting in the hot-rolled sample is significantly higher and bigger than that in the ECAPed sample, which further confirms that the ECAPed Ti-2Fe-0.1B alloy has better corrosion resistance.

Due to the high Cl^−^ concentration in the 3.5 wt.%, Cl^−^ can pass through the passivation film to reach the matrix, so that substrate and surface passivation layer forms a corrosion primary battery, and the matrix reacts rapidly with Cl^−^ in seawater, which is easy to cause the corrosion pitting [33]. According to the analytical results above, the corrosion resistance of ECAPed Ti-2Fe-0.1B alloy in 3.5 wt.% NaCl solution is better than that of a hot-rolled state. As it is well known, the ECAP method could reduce the grain size and increase the volume fraction of grain boundaries in the microstructure significantly. As the above results indicated that the grain has been significantly refined, the average grain size of the ECAPed decreased by 86% compared with hot-rolled samples. Furthermore, a large volume of dislocations is also introduced in grain interior and grain/subgrain boundaries of the ECAPed sample. These factors could increase the nucleation sites of the passivation film, which could promote the rapid formation and strong self-repairing ability of the passivation film on the surface, as well as increase the thickness of the passivation film [19,34]. Then, they can effectively protect the substrate from the attack of chloride ions in the solution and increase the corrosion resistance of the ECAPed titanium alloy. This is consistent with previous results reported by Sotniczuk et al. [32] and Yu Z et al. [25]. Generally speaking, the higher stored energy, the less stable the material, intergranular corrosion is easier to start and extend at HAGBs [17,33]. After the ECAP deformation, although the high stored energy can be formed with the decrease in grain size and the increase in dislocations and grain boundaries in the material, there is a much higher volume of LAGBs (which is shown in Table 1) in comparison with the hot-rolled state, which implies that intergranular corrosion can be inhibited by LAGBs even it happened at HAGBs. In addition, it is worth noting that the Ti-2Fe-0.1B alloy has a lower *i*_corr_ and superior electrochemical impedance performance compared to commercially pure titanium in 3.5 wt.% NaCl solution [18]. It seems like the addition of a proper amount of Fe can increase the corrosion resistance of titanium alloys [35,36], although the effect of Fe on corrosion behavior of titanium alloys still have controversy [37]. Similar conclusions have also been reported in the study of the Ti-6Al-xFe alloy in simulated body fluid solution by Lu et al. [35], and superior corrosion resistance than that of Ti-6Al-4V for Ti-Fe-O-N alloys have been reported in dental application [38].

## 4. Conclusions

In the present work, microstructure evolution, mechanical properties and corrosion behavior of hot-rolled and ECAPed Ti-2Fe-0.1B alloy were investigated. The main conclusions can be summarized as follows:

(1) Bimodal grain size distribution microstructure was fabricated by the hot-rolling process in Ti-2Fe-0.1B titanium alloy, which processes a good combination of strength (around 663 MPa) and ductility (around 30%).

(2) The high tensile strength around 854 MPa and good ductility around 15% were achieved after four subsequent passes of ECAP deformation as a result of the remarkable grain refinement of Ti-2Fe-0.1B alloy with the average grain size decreased to about 0.24 μm.

(3) Electrochemical experiments indicated that the ECAP deformation can also improve the corrosion resistance of the Ti-2Fe-0.1B alloy.

(4) The new low-cost titanium alloy after hot-rolling and ECAP could be used instead of Ti-6Al-4V in some industrial applications due to similar mechanical behavior and better corrosion resistance.

## Figures and Tables

**Table 1:** Microstructure and property parameters of Ti-2Fe-0.1B alloy in different states.

Sample States	Avg·GS (μm)	HAGB (%)	LAGB (%)	The Fraction of Grain in Different States (%)			Mechanical Behavior		
				Recrystallized	Substructured	Deformed	σ_0.2_ (MPa)	σ_s_ (MPa)	*A* (%)
Hot-rolled	1.72	66.94	33.06	24.7	63.1	12.2	457± 10	663 ± 2	30 ± 0.6
Annealed	3.48	81.41	18.59	83.2	11.5	5.3	445 ± 5	652 ± 4	24 ± 0.5
ECAPed	0.24	47.96	52.04	2.7	34.3	63	637 ± 8	854 ± 6	15 ± 0.4

**Table 2:** Several corrosion parameters of hot-rolled and ECAPed Ti-2Fe-0.1B alloy.

Sample	*E*_corr_/V	*i*_corr_/μA·cm^−2^	*E*_pit_/V	*i*_pass_/μA·cm^−2^	*R*/mm·a^−1^
Hot-rolled	−0.286	0.315	−0.052	0.82	0.0027
ECAPed	−0.261	0.053	0.065	0.11	0.0005

**Table 3:** Fitting electrochemical parameters of hot-rolled and ECAPed Ti-2Fe-0.1B alloy from experimental data by electrical equivalent circuit (EEC).

Sample	*R*_s_/Ω cm^2^	*CP*/10^−5^ S s*^n^* cm^2^	*n*	*R*_p_/10^5^ Ω cm^2^	Chi-Squared	*d*/nm
Hot-rolled	5.01	2.81	0.92	5.99	0.002176	1.97
ECAPed	8.27	2.60	0.93	16.36	0.000644	2.23
